# Medulloblastoma Presenting With Bilateral Internuclear Ophthalmoplegia of Abduction: A Case Report

**DOI:** 10.7759/cureus.95315

**Published:** 2025-10-24

**Authors:** Andrew Palmier, Sarah Grech

**Affiliations:** 1 Ophthalmology, Mater Dei Hospital, Msida, MLT

**Keywords:** bilateral internuclear ophthalmoplegia, clinical neuroanatomy, internuclear ophthalmoplegia, pediatric medulloblastoma, pseudo-internuclear ophthalmoplegia

## Abstract

Medulloblastoma is the most common paediatric high-grade central nervous system tumour. To our knowledge, this is the first case of medulloblastoma presenting with bilateral internuclear ophthalmoplegia (INO) of abduction. We describe a rare case of a 14-year-old girl presenting with bilateral abduction deficits and optic disc swelling. Neuroimaging revealed a midline posterior fossa tumour consistent with medulloblastoma compressing the dorsal pons and 4th ventricle. Following surgical resection and adjuvant therapy, ocular motility normalized. This case report highlights the features of a rare horizontal gaze palsy; bilateral posterior INO of abduction, resulting from the mass effect of a large medulloblastoma. Such a rare presentation requires a high index of suspicion and familiarity with neuroanatomy and neuro-ophthalmic examination in order to ensure correct diagnosis, prompt investigation, and treatment of the cause.

## Introduction

Horizontal gaze disorders are common clinical clues to brainstem pathology and can provide precise lesion localization when correctly interpreted. Among these, internuclear ophthalmoplegia (INO) is particularly characteristic and remains a hallmark of medial longitudinal fasciculus (MLF) dysfunction. Early recognition of this entity is essential, as it may signal underlying stroke, demyelination, or posterior fossa mass lesions.

INO is a classic brainstem gaze palsy caused by a lesion in the MLF, a paired white matter tract that coordinates conjugate eye movements by linking the abducens nucleus in the pons to the contralateral oculomotor nucleus in the midbrain. Disruption of this pathway results in impaired adduction of the ipsilateral eye during horizontal gaze, often accompanied by abducting nystagmus of the contralateral eye. Convergence may be preserved or variably impaired depending on lesion extent and location. The MLF lies dorsomedially in the brainstem; ventral to the cerebral aqueduct in the midbrain, and anterior to the fourth ventricle in the pons. Fibers within the MLF coordinate not only horizontal gaze via excitatory burst neurons but also vertical and torsional movements by integrating input from vestibular and ocular motor nuclei [1].

The hallmark of INO is adduction lag on attempted horizontal gaze, which may range from subtle slowing to complete inability to adduct beyond midline. The contralateral abducting nystagmus is thought to represent an adaptive overshoot driven by Hering’s law of equal innervation. When present, preserved convergence offers further diagnostic confirmation, although it may not always be reliable in mild or atypical cases.

An atypical variant was first described by Anton Lutz in 1923 and is historically referred to as posterior INO of Lutz. It is now more accurately described as reverse INO, pseudoabducens palsy, or INO of abduction. Clinically, it is characterized by an abduction deficit in the affected eye with preserved adduction and convergence. This presentation mimics a sixth nerve palsy, but key distinguishing features include: preserved abduction with the vestibulo-ocular reflex (VOR), possible contralateral adducting nystagmus, and normal pursuit and saccadic tracking, when assessed with tools like infrared oculography [2,3]. Anatomically, INO of abduction is thought to involve the abducens fasciculus, posterior pontine MLF, or paramedian pontine reticular formation (PPRF). The pathophysiology may relate to disrupted inhibitory projections to the medial rectus motoneurons or impaired coordination within the horizontal gaze network.

Although historically referred to as a “posterior” INO by Lutz, the term is now considered a misnomer, as the implicated fibers are not “posterior” in a neuroanatomical sense. Kommerell suggested the more accurate term “INO of abduction” to reflect the clinical phenotype rather than presumed anatomy [4]. The terminology originally proposed by Lutz should not be confused with Cogan’s classification of INO, in which the terms “anterior” and “posterior” refer to lesion location along the MLF; with anterior INO involving the rostral midbrain and resulting in impaired convergence, and posterior INO involving the dorsal pons, where convergence remains intact [1].

Although rare, INO of abduction has been described in association with multiple sclerosis, brainstem infarction, and neoplastic compression. Recognition of this atypical presentation is crucial, as it can direct localization to the dorsal pons near the floor of the fourth ventricle, particularly in the context of posterior fossa tumours, as demonstrated in our case. This article was previously presented as an oral presentation at the European Society of Ophthalmology (SOE) 2025 Congress in Lisbon, Portugal, on June 8, 2025.

## Case presentation

A 14-year-old Caucasian girl presented to the Emergency Department in view of a one-week history of reduced vision and binocular diplopia. This was preceded by a two-month history of headaches, intermittent vomiting, and generalised fatigue. Her past medical history and her family history were unremarkable. Snellen Visual Acuity (VA) on the right eye was 6/12-2 with no improvement on pinhole, and the VA on the left eye was 6/12-3, improving to 6/9 on pinhole. No relative afferent pupillary defect (RAPD) was observed. On examination, a horizontal gaze palsy was evident. Extraocular muscle movements (EOM) showed a limitation of abduction bilaterally with contralateral adducting nystagmus. Convergence was preserved, and abduction improved with the vestibulo-ocular reflex (VOR), suggesting a supranuclear cause. These findings were consistent with bilateral INO of abduction. Fundoscopy revealed bilateral optic disc swelling (grade 3 Frisen scale), while the remainder of her cranial nerve and neurological examination was normal.

Differential diagnoses at presentation included bilateral abducens nerve palsy due to raised intracranial pressure. The preserved convergence, intact VOR, and absence of significant esotropia in primary gaze helped differentiate this finding from a sixth nerve palsy and supported a diagnosis of bilateral INO of abduction, secondary to a structural lesion. An urgent computed tomography (CT) scan of the head revealed a relatively well-defined central cerebellar mass associated with perilesional oedema, causing effacement of the fourth ventricle and moderate dilatation of the third and lateral ventricles.

**Figure 1:**
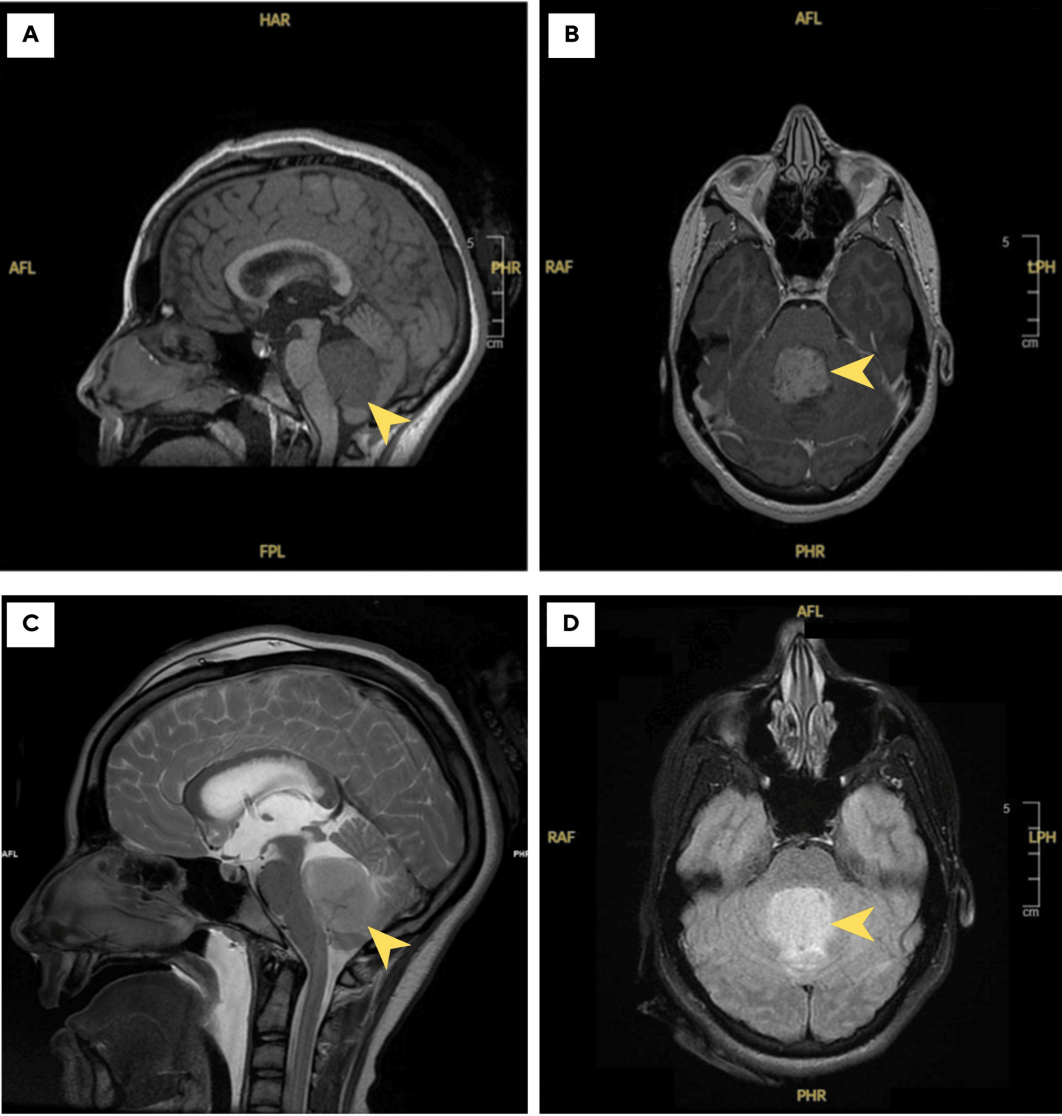
MRI images of the brain MR imaging showing a 42mm mass (yellow arrowheads) arising from the 4th ventricle, appearing hypointense to grey matter on T1w images (Sagittal scan A), with homogenous contrast enhancement (Coronal scan B) and hyperintense on T2w sequence images (Sagittal scan C) and with better delineation and increased hyperintensity on FLAIR sequence (Coronal Scan D).

The patient was started on intravenous dexamethasone and an external ventricular drain was inserted. Furthermore, a brain Magnetic Resonance Imaging (MRI) was performed and showed a 42mm mass arising from the 4th ventricle, appearing hyperintense on T2/FLAIR and hypointense to grey matter on T1 sequences with a mass effect on the cerebellopontine angle and surrounding vasogenic oedema. Radiological findings were highly suggestive of medulloblastoma (Figure 1).

The patient was transferred urgently to a specialised paediatric neurosurgical centre in the United Kingdom. She underwent excision of the tumour, craniospinal irradiation, and adjuvant chemotherapy. Immunohistochemistry indicated a molecularly defined subtype of Medulloblastoma, non-Wingless/non-Sonic Hedgehog (non-WNT/non-SHH). At the six-month follow-up, her visual acuity had returned to baseline, extraocular movements were full, and the bilateral INO of abduction had resolved completely. No recurrence or new neurological deficits were noted on surveillance MRI.

## Discussion

The coordination of horizontal gaze is a finely tuned neurological process that relies on both excitatory and inhibitory circuits within the brainstem. When a lateral gaze is initiated, the frontal eye field on one side activates the contralateral PPRF, which in turn stimulates the ipsilateral abducens nucleus [5]. This nucleus gives rise to two critical outputs: motor neurons that innervate the ipsilateral lateral rectus muscle to produce abduction, and internuclear neurons that cross the midline and ascend in the contralateral MLF to innervate the medial rectus subnucleus of the oculomotor nerve, producing adduction of the opposite eye. This coupled action enables conjugate horizontal gaze, allowing both eyes to move synchronously in the same direction [1,3]. A number of variants of INO have been described, varying in clinical presentation according to the segment of the horizontal gaze pathway which is affected (Table 1 ).

**Table 1:** Variants of internuclear ophthalmoplegia (INO), summary of clinical variants of INO, their distinguishing features, and lesion localization WEBINO: Wall-Eyed Bilateral Internuclear Ophthalmoplegia, WEMINO: Wall-eyed monocular internuclear ophthalmoplegia, MLF: medial longitudinal fasciculus.

Variant	Clinical Features	Typical Lesion Location
Classic INO	Adduction deficit with contralateral abducting nystagmus; convergence often preserved	MLF (pons or midbrain, depending on anterior vs posterior)
Anterior INO (Cogan)	Adduction deficit with *impaired convergence*	Rostral midbrain, near oculomotor nucleus
Posterior INO (Cogan)	Adduction deficit with *preserved* *convergence*	Dorsal pons (caudal MLF near abducens nucleus)
INO of abduction	Abduction deficit, contralateral adducting nystagmus, preserved convergence	Dorsal pons, abducens fasciculus, posterior MLF, possibly inhibitory pathway
WEBINO	Bilateral INO with large-angle exotropia in primary gaze	Bilateral MLF lesions, often midbrain
WEMINO	INO with ipsilateral exotropia	Unilateral MLF lesion (usually pontine)
One-and-a-half syndrome	INO + ipsilateral horizontal gaze palsy	MLF + ipsilateral PPRF or abducens nucleus
INO-plus syndromes	INO with skew deviation, trochlear palsy, or vertical gaze limitation	MLF + adjacent brainstem structures
WEBINO-plus	WEBINO associated with vertical gaze palsy or skew	Bilateral MLF + rostral midbrain
Pseudo-INO (myasthenia gravis)	Clinical picture mimicking INO but due to neuromuscular junction (NMJ) dysfunction	No structural lesion; autoimmune (NMJ)

Traditionally, the MLF has been conceptualized as a purely excitatory conduit linking the abducens and oculomotor nuclei. However, experimental data from primate and feline studies have provided compelling evidence for an additional inhibitory component within this system, aimed specifically at regulating the medial rectus motoneurons. Fibre degeneration and autoradiographic tracing techniques have demonstrated the existence of a direct, uncrossed inhibitory projection from neurons in the pontine reticular formation, specifically between the levels of the fourth and sixth cranial nerve nuclei, to the ipsilateral oculomotor nucleus. These fibres ascend adjacent to but separate from the MLF and terminate on medial rectus motoneurons. Functional studies have shown that stimulation of these pontine neurons can evoke monosynaptic inhibitory potentials in medial rectus motoneurons, even in animals with bilaterally destroyed MLFs. This suggests that the pathway operates independently of the classic MLF-mediated excitatory circuit and may serve to reduce tonic medial rectus activity during ipsilateral abduction, thereby facilitating smooth and coordinated lateral eye movements [6-9].

Disruption of this inhibitory system is hypothesized to underlie the phenomenon of INO of abduction. In this condition, patients present with an abduction deficit that mimics a sixth nerve palsy, but with preserved convergence and normal or compensatory adduction nystagmus in the contralateral eye. Unlike classical INO, which results from failure of adduction due to interruption of excitatory fibres in the MLF, reverse INO may reflect a failure to inhibit the ipsilateral medial rectus during attempted abduction. This leads to unopposed medial rectus tone, creating a false appearance of lateral rectus weakness [6,9].

In our case, neuroimaging demonstrated a midline posterior fossa mass arising from the fourth ventricle with compression of the dorsal pons and effacement of the fourth ventricular floor. This region contains the abducens nucleus, MLF, and the proposed inhibitory projections to the oculomotor nucleus; all of which are integral to horizontal gaze coordination [8]. Compression and displacement of these structures by the medulloblastoma, together with associated vasogenic oedema, likely caused disruption of both excitatory and inhibitory circuits, producing the observed bilateral INO of abduction. Similar mechanisms have been described in previous reports of brainstem tumours and pontine gliomas, causing either classical or reverse INO [10].

Medulloblastoma typically arises from the cerebellar vermis and extends anteriorly into the fourth ventricle, where mass effect and distortion of the dorsal pontine tegmentum can impair MLF conduction [11]. The reversibility of the INO following surgical decompression and adjuvant therapy supports a predominantly compressive rather than infiltrative pathophysiology. Comparatively, previously reported tumour-related cases of INO of abduction are rare, with most examples arising from intrinsic pontine gliomas or demyelinating plaques rather than extrinsic mass effect [3,9]. Our case adds to the literature by demonstrating that posterior fossa tumours such as medulloblastoma can produce this phenotype through mechanical distortion of dorsal pontine gaze pathways.

Clinically, distinguishing INO of abduction from an abducens nerve palsy is critical, as the former represents a supranuclear dysfunction with different prognostic and aetiological implications. The presence of preserved vestibulo-ocular reflexes and convergence, coupled with orthophoria in primary gaze and absence of significant esotropia, should raise suspicion for a diagnosis of INO of abduction.

## Conclusions

INO of abduction is a very rare eye movement disorder with an incidence of less than one-tenth as compared to classic INO. Recognition of this unusual but localizing ocular motor syndrome is clinically significant, as it may represent the first manifestation of posterior fossa pathology such as medulloblastoma. Its identification underscores the intricate interplay of excitatory and inhibitory control within the horizontal gaze network and the importance of precise neuroanatomical localization in neuro-ophthalmological diagnosis.
